# Primary anaplastic large cell lymphoma of the breast arising in reconstruction mammoplasty capsule of saline filled breast implant after radical mastectomy for breast cancer: an unusual case presentation

**DOI:** 10.1186/1746-1596-4-11

**Published:** 2009-04-02

**Authors:** Mona RY Bishara, Cathy Ross, Monalisa Sur

**Affiliations:** 1 Department of Anatomical Pathology, Henderson Hospital, 711 Concession Street, ON L8V13C, Hamilton, Ontario, Canada

## Abstract

**Background:**

Primary non-Hodgkin lymphoma (NHL) of the breast represents 0.04–0.5% of malignant lesions of the breast and accounts for 1.7–2.2% of extra-nodal NHL. Most primary cases are of B-cell phenotype and only rare cases are of T-cell phenotype. Anaplastic large cell lymphoma (ALCL) is a rare T-cell lymphoma typically seen in children and young adults with the breast being one of the least common locations. There are a total of eleven cases of primary ALCL of the breast described in the literature. Eight of these cases occurred in proximity to breast implants, four in relation to silicone breast implant and three in relation to saline filled breast implant with three out of the eight implant related cases having previous history of breast cancer treated surgically. Adjuvant postoperative chemotherapy is given in only one case. Secondary hematological malignancies after breast cancer chemotherapy have been reported in literature. However in contrast to acute myeloid leukemia (AML), the association between lymphoma and administration of chemotherapy has never been clearly demonstrated.

**Case Presentation:**

In this report we present a case of primary ALCL of the breast arising in reconstruction mamoplasty capsule of saline filled breast implant after radical mastectomy for infiltrating ductal carcinoma followed by postoperative chemotherapy twelve years ago.

**Conclusion:**

Primary ALK negative ALCL arising at the site of saline filled breast implant is rare. It is still unclear whether chemotherapy and breast implantation increases risk of secondary hematological malignancies significantly. However, it is important to be aware of these complications and need for careful pathologic examination of tissue removed for implant related complications to make the correct diagnosis for further patient management and treatment. It is important to be aware of this entity at this site as it can be easily misdiagnosed on histologic grounds and to exclude sarcomatoid carcinoma, malignant melanoma and pleomorphic sarcoma by an appropriate panel of immunostains to arrive at the correct diagnosis of ALCL.

## Introduction

Primary non-Hodgkin lymphomas (NHL) of the breast are uncommon. NHLs of the breast represent 0.04–0.5% of malignant lesions of the breast and account for 1.7% to 2.2% of extra-nodal NHLs [1,2]. Most primary cases are B-cell phenotype and are more common on the right side with only rare cases showing a T-cell phenotype. The primary lymphomas have been defined by the criteria of Wiseman and Liao, which have been followed by most of the authors with minor modification [3].

Anaplastic large cell lymphoma (ALCL) is a rare T-cell lymphoma typically seen in children and young adults. It has been described in numerous sites; however the breast is one of the least common locations. Secondary hematological malignancies after breast cancer chemotherapy have been reported in the literature [4,5]. Acute myeloid leukemia (AML) and myelodysplastic syndrome (MDS) are the common hematological disorders associated with breast cancer chemotherapy [5].

In this report we describe a case of ALCL of the left breast arising in reconstruction mammoplasty capsule of saline filled breast implant after radical mastectomy for infiltrating ductal carcinoma followed by post-operative chemotherapy twelve years ago.

## Report of a case

This is a 66 years old woman who had a left modified radical mastectomy in 1994 for infiltrating ductal carcinoma followed by post-operative chemotherapy. In 1999 she underwent breast reconstructive surgery with insertion of a saline filled breast implant. The implant was symptom free until March 2006 when the patient started to complain of pain at the site of the implant with deviation of the implant toward the axilla.

On examination there were no signs of infection, breakdown or ulceration but there was tenderness and some erythema. The implant was properly sized with grade II capsular contraction. There was no evidence of any mass along the mastectomy scar or any mass outside the capsule or axillary adenopathy. Mammogram and breast ultrasound did not show any evidence of recurrent malignancy but did show partial deflation of the implant with capsular contraction.

Taking into account the pain and the capsular contraction, the patient underwent capsulectomy with removal of the implant and insertion of a new implant. At the time of operation, an irregular mass with some necrosis was identified medially at 9 o'clock position. Intraoperative biopsy was taken and a frozen section was done. Frozen section showed large pleomorphic cells consistent with malignancy; hence the surgeon proceeded with excision of the whole mass with removal of the implant and the capsule on which a diagnosis of Primary ALCL was made.

Radiological studies of the chest, abdomen and the pelvis were clear. Bone marrow core examination was negative.

The patient was treated with three cycles of CHOP chemotherapy regimen and also received external beam field radiation. The patient is alive until the current date with no evidence of disease.

The excision specimen of the left breast capsulectomy specimen was fixed in 10% buffered formalin and sections from paraffin wax embedded tissue blocks were stained with hematoxylin & eosin (H&E).

Immunohistochemistry was performed using standard labeled Strep-Avidin-Biotin Technique (LSAB) using the following antibodies: LCA, AE1AE3, CAM5.2, EMA, CK7, CK20, 34BE12, Vimentin, CD20, CD79A, CD3, CD5, CD7, CD4, CD8, CD15, CD30, CD1A, CD21, CD23, ALK-1, Myeloperoxidase, CD117, EBV-LMP, Fascin, CD56, IgA, CD34, CD31, Factor VIII, S-100 protein, PNL2, Melanoma Cocktail, CD68, PLAP, Desmin, Alpha-1 Antitrypsin, Alpha-1 Antichymotripsin, Factor X111A.

Molecular analysis was performed for B and T cell gene rearrangement using standard polymerase chain reaction (PCR).

**Figure 1:**
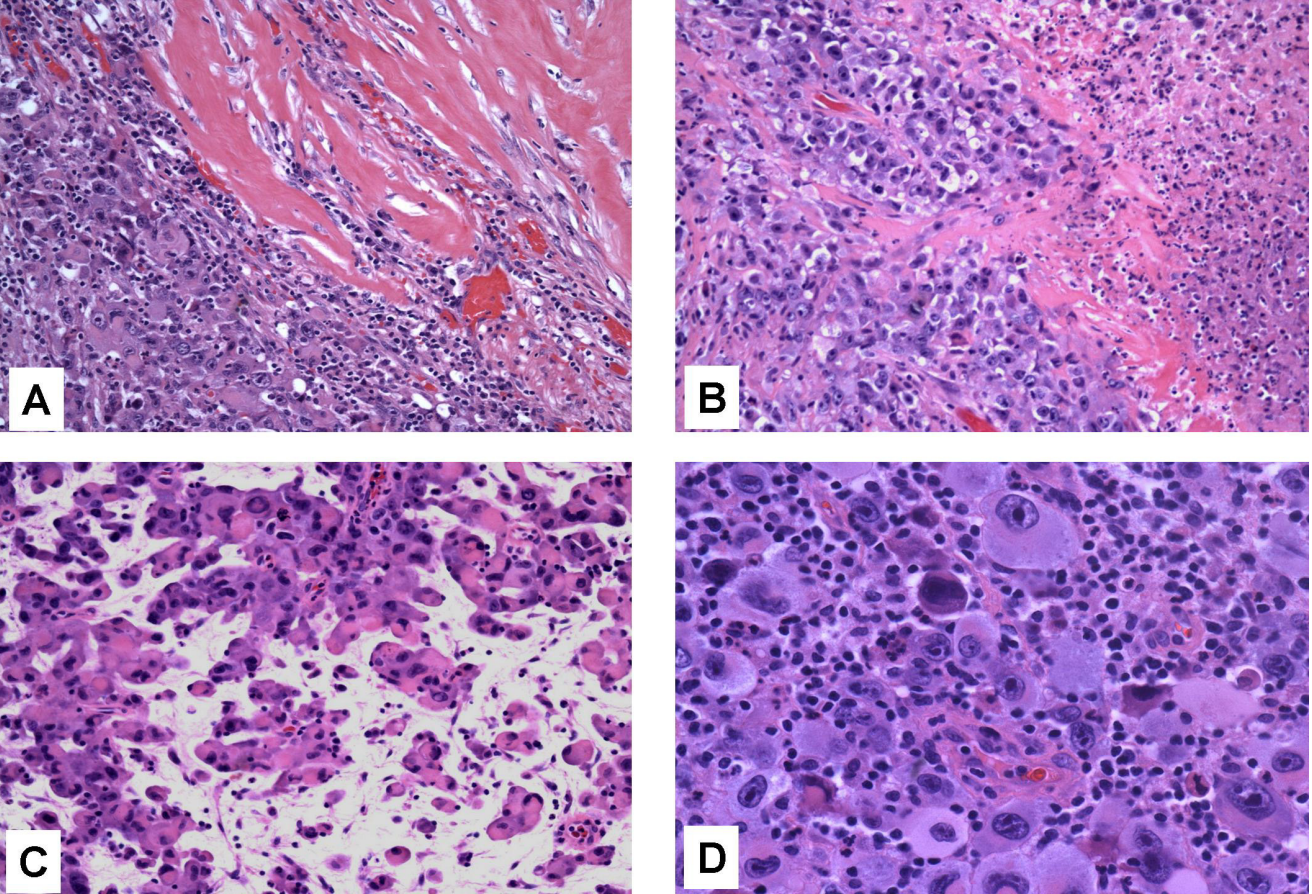
**A, B, C and D**. Pleomorphic large cells infiltrating the implant capsule in solid sheets. H&E ×100 (A) and showing areas of necrosis H&E × 200(B), Pleomorphic large cells in an edematous background present singly and in small clusters mimicking a carcinoma. H&E × 200 (C), Pleomorphic cells with anaplastic nuclei with prominent nucleoli in a background of inflammatory cells. H&E ×400 (D).

Microscopic examination of the H&E stained sections showed diffuse sheets of large atypical cells having bulky eosinophilic to foamy/vacuolated, amphophilic cytoplasm. The nuclei were large and pleomorphic with vesicular chromatin and prominent one to two nucleoli. Numerous pleomorphic multinucleate giant cells, binucleate cells, mononucleate cells and cells with horseshoe-shaped and reniform nuclei with prominent nucleoli were present (Figs 1A, B, C, D). There was brisk mitotic activity and areas of necrosis. The tumor cells were infiltrating adipose tissue but did not demonstrate angiotropism or angiodestruction. The background showed numerous histiocytes, eosinophils, plasma cells and neutrophils. Based on the morphological features, the differential diagnosis included sarcomatoid/anaplastic carcinoma, malignant melanoma, pleomorphic sarcoma NOS and hematological neoplasms such as Hodgkin's lymphoma and the rare ALCL.

**Figure 2:**
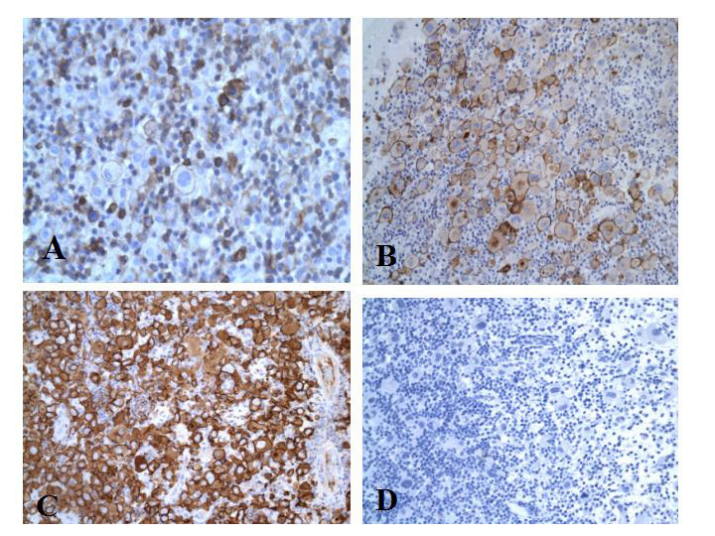
**A, B, C, and D**. Tumor cells positive for immunostain for LCA × 200 (A), Tumor cells positive for immunostain for EMA × 200 (B), Tumor cells positive for immunostain for CD30 × 200 (C), Tumor cells negative for immunostain for ALK-1 × 200 (D).

Immunohistochemistry demonstrated that the large atypical neoplastic cells were focally positive for LCA (Fig 2A) and EMA (Fig 2B) and strongly positive for CD30 (Fig 2C) and Vimentin. The tumor cells were negative for all other markers including ALK-1 (Fig 2D). The background reactive lymphocytes showed positivity for T-cell markers with few cells showing positivity for B-cell markers. The histiocytes stained positive for histiocytic markers.

Molecular analysis by PCR on DNA extracted from the paraffin embedded tissue showed a monoclonal T-cell gene rearrangement for the joining and variable regions of the T-cell gamma receptors with V-gamma I and V-gamma II primers.

Based on the histology and ancillary tests a diagnosis of primary ALK negative ALCL, T-cell phenotype was made on the specimen and other entities considered in the differential diagnosis were excluded.

## Discussion

Primary NHL of the breast is rare and represents 0.04% to 0.5% of all malignant breast tumors and accounts for 1.7% to 2.2% of extra-nodal NHLs [1,2]. Most cases are of B-cell phenotype and are more common on the right side with rare cases showing T cell phenotype. The primary lymphomas have been defined by the criteria of Wiseman and Liao as follows:

1. Technically adequate specimen.

2. Mammary tissue and lymphomatous neoplasms in close association.

3. No evidence of concurrent wide spread disease (as evidenced by clinical presentation, radiologic studies and in more recent cases magnetic resonance imaging study).

4. No prior diagnosis of extra mammary lymphoma [3].

ALCL accounts for approximately 3% of adult non-Hodgkin lymphoma and involves both lymph nodes and extra-nodal sites. Extra-nodal ALCL has been found in the skin (21%), soft tissue (17%), bone (17%), lung (11%), and liver (8%). Involvement of the breast is rare in primary ALCL [6].

Two clinical forms of the disease have been described, the more common systemic form accounts for 3% of all adult non-Hodgkin lymphoma, and the less common cutaneous form accounts for 25% of all primary cutaneous lymphomas. Although both forms express CD30, systemic ALCL is the only one that expresses ALK-1 and shows the characteristic balanced translocation; t [2,5] in the majority of cases [6,7].

There are a total of eleven cases of primary ALCL of the breast previously reported in the literature [8-17]. Eight of these cases occurred in proximity to breast implants; five in relation to silicone implant, three in relation to saline filled implant similar to our case, with previous history of breast cancer in four of the eight cases. These four cases were treated surgically with adjuvant postoperative chemotherapy given in only one case and none of these cases had received radiotherapy [8-11,15].

The clinical presentation of ALCL is usually a mass, which is sometimes painful [18]. In contrast, patients who developed breast lymphoma in proximity to breast implant usually presented with implant-related symptoms with or without a mass lesion [9]. Our case, presented with implant related symptoms without mass lesion. Therefore, these cases emphasize the need for careful pathologic examination of tissue removed for implant related complications.

Since eight out of the eleven cases of primary ALCL occurred in relation to breast implants, it is possible that there is a relation between the nature of the implant and the risk of occurrence of lymphoma. Despite early reports that silicone was biologically inert in human tissue, the safety of breast implant for augmentation mammoplasty has been a subject of much discussion with respect to local and systemic complications, including carcinogenic effects. An Independent panel of US scientists convened and concluded that silicone breast implant do not cause any systemic disease, but these implants can leak and rupture, causing local problems such as scarring, infection and disfigurement. [8,19,20]. The four cases of ALCL of the breast that occurred in proximity to a silicone breast implant among more than one million women with breast implants, raises the possibility that the occurrence of lymphoma in those women may not be related directly to the implant and other unknown factors may have contributed to the development of lymphoma in these patients [8]. However, in a recent study from Netherlands, eleven cases of primary breast ALCL were described over a period of 17 years where five out of the eleven cases occurred in relation to silicone breast implant performed for cosmetic reasons. There was no history of cancer, chemo or radiotherapy. In this study the odds ratio of ALCL associated with breast prostheses was 18.2, however the absolute risk was very low due to the rare occurrence of ALCL of the breast [21].

The risk of developing a primary hematological malignancy in patients with breast cancer treated with post-operative chemotherapy is approximately 1.5% and the cumulative risk increases by 0.25–1% for the first 8 years after treatment. There are several reported cases of AML and MDS arising in patients with breast cancer treated post-surgery with chemotherapy and radiotherapy [5]. There is one case series of six patients developing secondary hematological malignancies after systemic chemotherapy for breast cancer of which two developed AML, one developed MDS, and 3 developed NHL (Diffuse large B cell lymphoma, Angio-immunoblastic T cell lymphoma and Mantle cell lymphoma respectively in nodal sites) [4,22]. There are random case reports on occurrences of secondary hematological malignancies other than AML and MDS [22,23]. In contrast to AML, the association between hematological malignancies and the administration of chemotherapy has never been clearly demonstrated. In this particular case, the ALCL is present at the site of the saline filled breast implant and the patient underwent treatment with adjuvant chemotherapy post-surgery for infiltrating ductal carcinoma 12 years ago.

ALCL shows a spectrum of morphologic features depending on the histologic types. To date three distinct histologic types have been described. These include the common, lymphohistocytic and small cell variant. Other less recognized descriptive subtypes include neutrophils rich, Hodgkin like and sarcomatoid variants [7]. Common to all these subtypes, is the presence of the characteristic hallmark cell, which is a large atypical cell with abundant cytoplasm and eccentric horseshoe shaped or reniform nuclei. These cells often have an eosinophilic region near the nucleus and prominent central nucleolus [6]. Our case could be best classified as lymphohistocytic subtype. Given the patient's previous history and present histology, it is important to exclude sarcomatoid carcinoma, malignant melanoma and pleomorphic sarcoma by an appropriate panel of immunostains to arrive at the correct diagnosis of ALCL, as this is a rare a rare entity and can be easily misdiagnosed.

Preliminary data shows that ALK-1 positivity is one of the most important predictors of prognosis in ALCL and is associated with favorable prognosis. In the eleven primary cases of ALCL, two cases were ALK-1 positive, [12,13] six were ALK-1 negative and in the remaining three cases the result of ALK was not known [9,11,14-17]. Our case was ALK negative and by molecular analysis demonstrated a T-cell phenotype. Due to the small number of cases identified in the breast, together with the limited follow up data and the unknown status of ALK-1 reactivity in some of the cases, it is difficult to predict the course of primary CD30 positive ALCL of the breast.

## Conclusion

In conclusion, we describe a case of primary ALK negative ALCL arising in the site of saline filled left breast implant 12 years after the patient underwent modified radical mastectomy followed by adjuvant chemotherapy for infiltrating ductal carcinoma of the breast. It is still unclear whether chemotherapy and breast implantation increases risk of secondary hematological malignancies significantly. However, it is important to be aware of these complications and need for careful pathologic examination of tissue removed for implant related complications to make the correct diagnosis for further patient management and treatment. It is therefore important for surgical pathologist to be aware of this entity at this site, as it can be easily misdiagnosed on histologic grounds.
